# Using energy budgets to combine ecology and toxicology in a mammalian sentinel species

**DOI:** 10.1038/srep46267

**Published:** 2017-04-07

**Authors:** Jean-Pierre W. Desforges, Christian Sonne, Rune Dietz

**Affiliations:** 1 Department of Bioscience, Arctic Research Centre, Aarhus University, Frederiksborgvej 399, PO Box 358, DK-4000 Roskilde, Denmark

## Abstract

Process-driven modelling approaches can resolve many of the shortcomings of traditional descriptive and non-mechanistic toxicology. We developed a simple dynamic energy budget (DEB) model for the mink (*Mustela vison*), a sentinel species in mammalian toxicology, which coupled animal physiology, ecology and toxicology, in order to mechanistically investigate the accumulation and adverse effects of lifelong dietary exposure to persistent environmental toxicants, most notably polychlorinated biphenyls (PCBs). Our novel mammalian DEB model accurately predicted, based on energy allocations to the interconnected metabolic processes of growth, development, maintenance and reproduction, lifelong patterns in mink growth, reproductive performance and dietary accumulation of PCBs as reported in the literature. Our model results were consistent with empirical data from captive and free-ranging studies in mink and other wildlife and suggest that PCB exposure can have significant population-level impacts resulting from targeted effects on fetal toxicity, kit mortality and growth and development. Our approach provides a simple and cross-species framework to explore the mechanistic interactions of physiological processes and ecotoxicology, thus allowing for a deeper understanding and interpretation of stressor-induced adverse effects at all levels of biological organization.

Current and classical approaches in ecotoxicology typically rely on regression modelling and/or summary statistics in order to describe observed effects in terms of EC_50_, no observable effect concentration (NOEC), etc. The major problem with the descriptive nature of classical approaches is the ignorance of interaction between ecology and toxicology as well as the lack of insight gained into the processes underlying toxic effects^1^,^2^. Other major shortcomings include the inability to incorporate temporal measurements or chronic exposure to multiple stressors, difficult interpretation to other species, no consideration of time dependency of exposure and effects, and no consideration of the biological background or relative importance of toxicity endpoints^1^.

Process-based approaches that can be used to further our understanding of the physiological implications of toxic exposures are attractive alternatives to traditional approaches in ecotoxicology. The conservation laws of mass and energy provide a basis for bioenergetics models, such as dynamic energy budget (DEB) theory, that study the flow of energy through living organisms as it relates to physiological processes such as growth, development, reproduction and maintenance^3^ (see Fig. 1). DEB theory resolves many of the shortcomings of descriptive toxicology through the implementation of a mechanistic-based framework that accounts for interactions between animal ecology, toxicology, and metabolic processes throughout the entire lifecycle of an organism. In this context, toxicants exert their effect on model parameters which in turn alters animal physiology and life-history output over time^4^,^5^. This can also allow predictions to be made outside of the conditions tested in experiments^2^, a notably important feature for marine wildlife being exposed to critally high levels of environmental toxicants^6^,^7^,^8^.

DEB theory is well established in theoretical biology as an approach to describe simple rules for metabolic organization. DEB has been used extensively to describe and predict life histories under different natural scenarios^9^,^10^ and under toxicant stress^1^,^5^,^11^. The generic rules of DEB apply to energy flow in all organisms, and such models can be adapted and applied to any species. Nonetheless, the DEB framework as applied in the standard model contains complex mathematics and parameterization that requires extensive species-specific data^3^. Simplified versions of the standard DEB model as well as free tools to help parameterize the model have been developed to aid in user implementation, including ‘the scaled standard model^12^ and ‘DEBtox^13^. More recently, the ‘DEBkiss’ model has been developed to allow easier implementation and understanding of DEB through fewer and more easy to understand equations and parameterization^4^. The applicability of DEBkiss to model life history and toxicology has been established in several organisms, though to date these have focused only on invertebrate organisms like snails, nematodes and crustaceans used in standard laboratory toxicity testing^11^,^14^,^15^.

The mink (*Mustela vison*) is a widespread carnivore abundant in temperate aquatic ecosystems. Mink inhabit coastal areas, have restricted home-ranges, and as fish-eating mammals they typically occupy high trophic levels and bioaccumulate local pollutants^16^. For these reasons the mink has been recognized as an ideal sentinel species to address integrative issues of exposure and effect to environmental toxicants^17^,^18^. Indeed, the mink has been used extensively as a classic model in mammalian toxicology for effects of many toxicants on various health endpoints, including reproduction and growth^19^,^20^,^21^. The usefulness of captive mink as a predictive model in toxicology to humans and other hard to study wildlife species is well established based on similarities in physiology and responses to toxic substances and diseases^20^.

In this study we use DEBkiss to model the life history and effects of toxicant stress in the mink as a mammalian sentinel species in environmental health. We used published literature on growth and reproduction in captive mink to parameterize a version of the basic DEBkiss that we have adapted to a mammalian lifecycle. The purpose of this study was to generate a baseline DEB model for mink, a classic model organism in mammalian toxicology, in order to assess the effects of dietary exposure to environmental toxicants on growth and reproduction. More specifically, we attempt to derive the targeted mode of action of these toxicants using pattern-oriented analysis of the output of our DEBkiss model. Ultimately, we hope this generic model may be used to interpret and predict other experimental stressors, individually and in combination, to captive and wild mink as well as other wildlife species.

## Results

### Growth and ingestion rate

The fit of the model to the growth data of female mink is shown in Fig. 2 and the estimated parameter values are presented in Table 1. Our simplified DEB model captured the observed von Bertalanffy growth pattern in mink and parameters were efficiently fitted as judged by their small standard deviations. Though body size data was only available for the first 200 days, the model predicted an ultimate body size well within the range observed in mink^22^,^23^,^24^.

Further validation of our DEBkiss model can be assessed through predictions of mass flow to processes other than growth. One such process is food ingestion rate since the assimilation flux *J*_*A*_ can be compared to actual ingestion data measured in grams dry weight per day. It should be stressed that no one study had data on growth, reproduction and ingestion, and such data were derived from multiple sources in available literature and represent animals at different ages, breed, life history and housing conditions. Furthermore, the assimilation flux represents assimilated food weight, which is different from ingested food weight measured in the literature. Nonetheless, this comparison allows a rough estimate of the representativeness of the model parameters. Ingestion rates for farmed female mink (selected for high feed efficiency) fed ad libitum ranged between 50 to 75 g dw/day^25^. Our model predicted an assimilation flux in adults around 35 g/day, which assuming a generic digestion efficiency of 80%, would result in an ingestion rate of approximately 44 g/day. Given the variety of factors and measurement errors that differ between studies, including the fact that farmed mink are genetically bred for high weight gain, our predictions reasonably fit the ingestion data. Increasing the ingestion rate in the model without increasing ultimate body size would require increasing the specific assimilation and maintenance rates (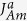 and 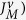, with direct consequences on other processes.

### Reproductive performance

To further assess the model we also looked at the 1-κ branch of energy allocation. The model predicted a first litter size of 5.6 kits, fitting very well within the general range observed in mink (3–9) and more specifically the average of 5–6 kits seen in the toxicology literature^23^,^26^,^27^. This model fit was achieved using a scaled functional response during reproduction (*f*_R_) of 2.52; in other words, this provided two and half times more assimilated mass to the 1-κ branch, or an additional 10 g/day of mass flux. This additional influx of energy during reproductive periods provided the necessary means to fuel the extra costs associated with embryo and milk production, while keeping adult body weight unaffected. The model results correspond with observations in captive mink where dam feed intake increased almost 3-fold during a four week nursing period while dam weight did not significantly change^22^. This aspect of reproductive biology is unique to mammals that have energy intensive lactation periods that often require additional feeding rather than draining current reserves.

All the subsequent litters after the first were predicted to produce 6.8 kits (Fig. 2). The increased reproduction rate after the first litter occurred because adult mink no longer invested in maturation and therefore had an entire year to accumulate energy in the reproduction & supply buffer (RS buffer) before the next reproductive cycle. Unfortunately, we could not locate any lifetime reproductive output studies in mink to compare cumulative reproduction rates. However, it is common in mammals for reproductive performance to increase as the mother gains experience, and then plateau until senescence at older ages^28^. We have not attempted to add the metabolic consequences of ageing and senescence in our model as we were interested in growth and reproduction at younger ages, however, such applications of DEB do exist^29^ and could help describe age-related reproductive performance.

### Toxicant stress and physiological modes of action (pMoA)

Understanding lifelong accumulation and the associated cumulative effects of toxicants is crucial to quantifying individual and population-level consequences of anthropogenic chemical exposure. DEB applications in toxicology can be used to predict the effect of toxicant exposures on particular physiological processes; conversely, information on growth and reproduction at different toxicant exposures can be used to identify which pMoA is most likely targeted. It is this pattern-oriented analysis that we used here to assess how PCBs, a major toxicant of interest, affected growth and reproduction in mink.

**Figure 1:**
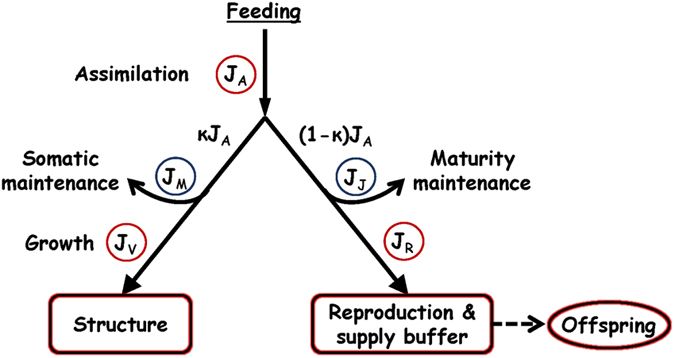
Simplified schematic diagram of mass flow in the mink DEB model. Toxicant stress on individual parameters are highlighted with colour: blue = increase, red = decrease. Description of the DEB parameters are provided in Table 1.

**Figure 2:**
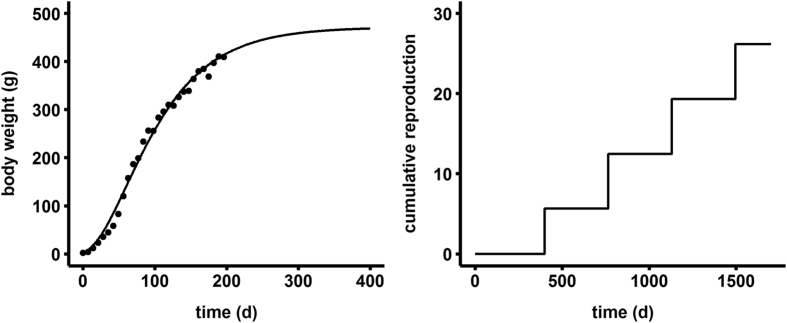
Fit of the DEB model to growth and reproduction data from captive mink fed ad libitum^47^.

A schematic representation of processes influenced by toxicant stress and detailed descriptions of affected parameters and pMoA are presented in Fig. 1 and Table 2. Model parameters were estimated by fitting to hepatic ∑PCB levels and growth and reproductive data from dietary exposed mink^27^. Average observed hepatic ∑PCB levels were 0.015, 0.092, 0.46 and 0.18 μg/g ww for the control and three exposure groups (0.25, 0.5 or 1.0 μg/g ww PCB) in 42 day old kits, and 0.12. 0.63, 0.96 and 1.50 μg/g ww in adult female mink after weaning their kits. Our toxicokinetic model, using an estimated BCF value of 3.33 ± 0.32, predicted internal ∑PCB concentrations in kits and adult female mink for the four diet groups of 0.0017, 0.088, 0.18 and 0.41 and 0.0096, 0.47, 0.93 and 1.81 μg/g ww.

**Figure 3:**
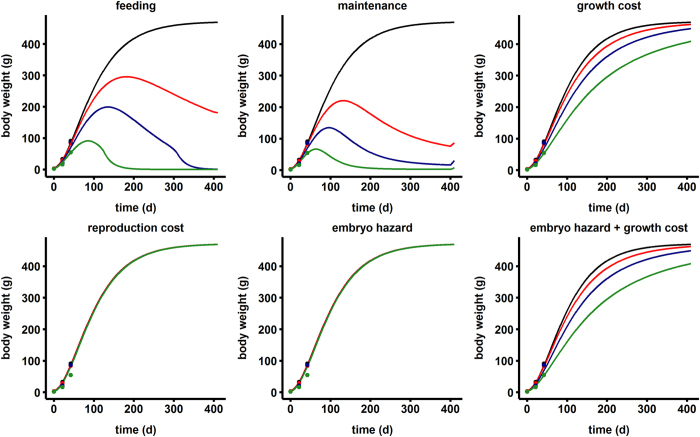
Fit of various physiological modes of action applied to the mink DEB model for growth in captive mink fed increasing doses of a PCB contaminated diet. Lines represent model fits for the different exposure groups: control (black), 0.25 μg PCB/g ww feed (red), 0.50 μg PCB/g ww feed (blue), 1.0 μg PCB/g ww feed (green). Growth was monitored only to day 42^27^.

**Figure 4:**
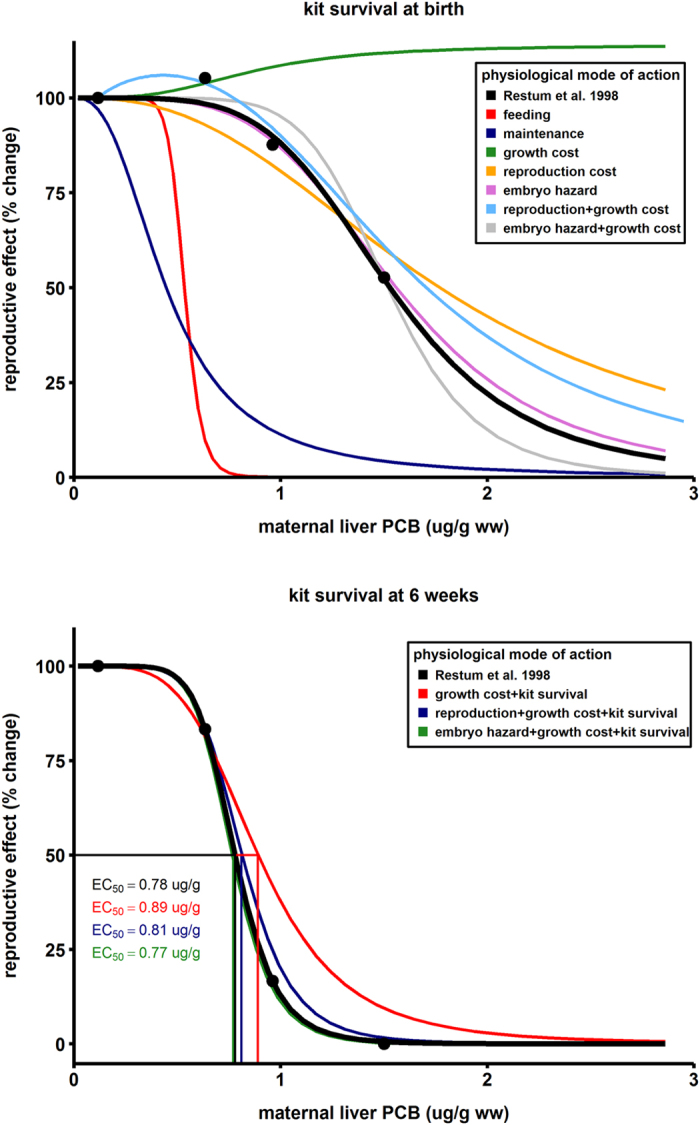
Modeled dose-responses for reproductive toxicity in captive mink fed PCB contaminated diets using different physiological modes of action (pMoA). The top panel shows the fit for all the pMoA for the effect of PCBs on kit survival measured at birth. The bottom panel shows only the best fitting pMoA to the effect of PCBs on kit survival 42 days post-parturition. PCB concentrations are hepatic levels measured in mothers and points correspond to empirical results^27^.

The effects of the four exposure doses on the body size and reproduction rate of mink as modelled by stress on different pMoA are displayed in Figs 3 and 4, respectively. The model was fitted to the observed data by estimating the tolerance parameter (*C*_*T*_, *C*_*Th*_) and the no-effect concentration (NEC) (Table 2). While decreased feeding/assimilation as well as increased somatic maintenance costs captured PCB-induced growth retardation for the first 42 days of the mink lifetime, these pMoA resulted in long lasting and severe/fatal effects on growth and adult body weight that were not observed in the data. Though effects on early growth are documented in many studies^23^,^26^,^27^, little evidence exists to suggest these continue so drastically into adulthood as predicted in the models for these pMoA. Furthermore, due to the interactions inherent in the model, the severe reductions in body size caused drastic reductions in reproduction rates that were not seen in the observed data (Fig. 4). These patterns therefore suggest two possible scenarios. First, assimilation and somatic maintenance are likely not the target pMoA for PCBs in mink. The alternate explanation is increased sensitivity in kits and/or developed tolerance in adults such that PCB effects are transient and occur only in young animals for these pMoA. Indeed, parental and developmental tolerance to toxicant effects is a confirmed phenomenon^30^,^31^. Under this hypothesis, the suppressive effect on body size would cease in juveniles and adults would ultimately recover and reach ‘normal’ body size prior to first reproduction. Deciphering between scenarios would require more weight measurements for exposed mink throughout their lifetime.

Increased growth costs is a more likely pMoA as the effect on growth was consistent with short and long term observations of body size (Fig. 3). The model predicted that this pMoA actually increases reproductive output in exposed animals due. This effect is a result of the important influence of body size (*L*) on energy utilization rates (Fig. 4); specifically, maturity maintenance scales with *L*^3^ such that costs are reduced in smaller animals (Table 1). The mismatch with reproductive observations despite capturing growth effects suggests that growth costs are not the sole target pMoA. Stress on reproductive costs and embryo hazard were ruled-out as sole pMoA as these had no influence on growth (Fig. 3). Of the two reproductive stressors, embryo hazard (i.e. embryo mortality) fit the reproductive dose-response data better (Fig. 4). Ultimately, to capture the stress influence on growth as well as reproduction it was necessary to combine pMoA. The best fitted model to observed data included the combined effects of growth costs and direct toxicity to the embryo.

Survival of newborns for the first 42 days (i.e. litter size at six weeks post-parturition) was also measured^27^ and we used the data to generate a dose-response curve reported as percent change in reproduction. Given the unlikely target of PCBs against assimilation and maintenance pMoA, we did not include these in this six-week kit analysis. To capture the additional risk to the newborn kit we included the addition of PCB induced kit mortality, modeled via a survival hazard in newborn kits (Table 2). Growth costs combined with kit mortality under-predicted effects at higher exposures, while the inclusion of reproductive costs or embryo hazard resulted in excellent predictions of the dose-response curve (bottom panel in Fig. 4). The EC_50_ based on hepatic PCB concentrations in the mother of observed data was 0.78 ± 0.002 μg/g ww and our best DEBkiss model predicted 0.77 ± 0.03 μg/g ww. Using DEB theory to model toxicant stress proved to be a unique and invaluable approach to not only accurately model toxicant accumulation and effects, but also to identify the most likely mode of action based on pattern-oriented analyses.

## Discussion

DEB theory provides a comprehensive framework with which to model animal physiology, life history and, when combined with other models, population dynamics. To date, most applications of DEB theory have focused on marine invertebrates and fish, though a wide and growing library of species is being incorporated into a DEB database (http://www.bio.vu.nl/thb/deb/deblab/add_my_pet/index.html). Although many mammalian species are included in this database thanks to parameter estimation software (DEBtox), there are few published studies validating the use of DEB for mammals. In this respect, we provide a timely development of a unique DEB model specific to the mammalian lifecycle that can predict individual and population relevant growth and reproduction parameters. Though parameterized using data from captive mink, our model is directly applicable to wild mink as well. Not only is our approach simple and easily understandable, it is generally applicable to other mammals with slight modifications specific to species biology.

Our incorporation of toxico-kinetics and –dynamics into a simple animal energy budget provided a unique approach to identify possible physiological modes of action of environmental toxicants. Our model predicted that dietary toxicant exposure (primarily PCBs) in mink affected embryo mortality, kit mortality and growth costs. While increased growth costs caused a delayed attainment of asymptotic body size over the first few years, the effects on reproduction and kit survival occurred throughout the mink’s lifetime. Extrapolating our model to the full eight year lifespan for captive mink^17^ reveals a reduction in average cumulative offspring production of 53% (20.9 kits), 96% (1.6 kits) and 100% (0 kits) for the three exposure groups relative to the unexposed control (44.8 kits). Modelled hepatic PCB concentrations after 8 years for the three exposure groups were 0.77, 1.66 and 3.33 μg/g ww, equivalent to 15.40, 33.20 and 66.60 μg/g lipid using a typical 5% lipid content for liver^23^. Such drastic effects on reproduction would obviously impact population dynamics over time leading to substantial reductions in population growth at these exposure levels. Environmental exposure to toxicants have been linked to declines in populations of wild mink near industrial point sources and those consuming highly contaminated fish^17^,^21^. Similar population-level effects have occurred in other marine species exposed to environmental toxicants, including otters^32^, seals^32^,^33^ and cetaceans^6^,^34^. Similar to our model findings, experimental and field studies with mink and other species have identified reproduction and development as sensitive targets for PCBs and related organic pollutants. Reproductive impairments have been linked to hormone and oestrous cycle imbalances, degeneration of the placenta and trophoblast, uterine occlusions and other reproductive lesions, and foetal death and resorption^17^,^19^,^21^,^35^,^36^. Specific targets of developmental toxicity and growth effects are more difficult to pinpoint, but may be linked to toxicant induced endocrine disruption, including effects on thyroid hormones and vitamin A^17^,^35^. Effects on assimilation and maintenance have also been associated with exposure to environmental toxicants, for instance via increased searching or handling time for prey, reduced digestion efficiency and costs of tissue lesion repair and immune defenses^11^,^17^,^37^,^38^. Altogether, empirical findings of PCB toxicity support the physiological modes of actions revealed by our model, further validating the use of DEB in toxicology and environmental risk assessment.

We have focused our attention on PCBs in the current implementation of our DEB model because these were determined by empirical studies to be most important in the mink toxicity studies. Although we use PCB concentration as our exposure metric, in reality the fish in the feeding study were taken from the polluted Lake Huron and contain the environmental mixture of toxicants found in that ecosystem^27^. Our study therefore represents the cumulative impact of all these chemical compounds, referenced through PCB concentrations. While it is impossible to tease out individual toxicant effects in this mixture based feeding study, DEB models are well suited to the study of multiple stressors, such as chemical mixture effects, using data from controlled exposure studies. In such models, the bioaccumulation of each toxicant is modelled separately via separate toxicokinetic sub-models and then individual effects on specific metabolic processes can be combined to determine the overall effect on growth and reproduction over the organisms entire lifespan^39^,^40^. Similarly, toxicant stress can be combined with other environmental stressors using the same modelling approach thanks to the fundamental and mechanism-based influence of different stressors on metabolic processes in DEB theory. Such an approach is ideal to describe the combined effects of high contaminant exposure and food stress for a species like the polar bear (*Ursus maritimus*) facing reduced food availability and quality due to sea-ice changes in a warming climate.

Although DEBkiss has been shown to work well in many invertebrate species^11^,^14^,^15^, we believe this is the first application of DEBkiss to a mammalian species. This is likely because of the novelty of DEBkiss as well as the lack of a ‘reserve’ compartment as used in standard DEB theory. The implementation of a reserve compartment can be intuitive for mammals that store large amounts of energy in adipose tissue, such as seals, whales and bears. The few DEB studies on mammals indeed include a reserve compartment which mainly takes the form adipose tissue^41^,^42^,^43^. Lipid reserves in these models were used to predict growth, reproduction, survival and pollutant toxicokinetics under different environmental or physiological scenarios. In summary, lipid reserves and associated energy density strongly predicted individual fitness. While our simplified DEBkiss model eliminates the reserve compartment and its associated dynamics, we do implement long term energy storage via the RS buffer placed in the 1-κ branch. This not only reduces the number of parameters in the model, but also makes away with rules for reserve dynamics. This simplification of energy storage dynamics appears to work well for the mink and various invertebrate species, but further work is needed to validate its use for other mammals.

Our DEB model is an obvious simplification of the complex metabolic processes occurring during the mammalian lifecycle. For instance, many aspects of mammalian reproduction, including embryo development and lactation, were simplified in order to reduce model complexity in this proof of concept study. With our simple mammalian model now validated, future work should focus on developing more realistic and complex sub-models for embryonic development and the post-parturition period where offspring rely completely on milk from the mother. Ageing and its metabolic consequences has been defined in a DEB context and should also be explored in future iterations of this and other models interested in full lifetime and population models^29^,^44^. Though we accurately modelled PCB accumulation in mink, a more complex toxicokinetic model could be adapted by integrating embryo development and lactation transfer more discreetly and by incorporating more physiologically based approaches.

The main uncertainty in the current parameter set is the value of κ and the scaled feeding parameter (*f*). We used the typical κ value of 0.8, though values for different species can range widely and models can be fit to empirical data using different κ values by adjusting other parameters (e.g. 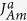)^14^. Decreasing κ increases mass flux to maturation and reproduction, thus replenishing the RS buffer much faster. However, information is lacking to confidently select the most realistic value for κ. We use a maximal value of 1 for *f* to capture the ad libitum food intake in captive feeding studies; the true value is a function of food density, the specific feeding rate and the specific searching rate^4^. These values would need further thought for models of wild animals experiencing temporal changes in food availability.

The parameter estimates for our model were limited by availability of data for mink life history parameters. Compared to well-studied invertebrates and laboratory rodents, mink are relatively long-lived and therefore more demanding and difficult to house and handle for long-term studies. Consequently, information on lifelong reproductive output and long-term toxicity studies are lacking for this species (and many other mammals). For existing toxicity studies, data are often only reported for growth or reproduction, not both, or only for a short period. More complete reporting of body size and reproductive output over time, coupled with measures of food ingestion rates and/or respiration rates, would facilitate implementation and validation of future DEB models.

The use of DEB approaches to describe standard laboratory toxicity testing is increasing, but we hope our modelling framework here encourages further development within this field to new mammalian species and wildlife. This type of process- or mechanism-based modelling is advantageous to simple descriptive dose-response curve fitting due to the inter-connectedness of parameters resulting in feedbacks and interactions between physiological processes. Such interactions are intuitive in a system with a finite energy supply and different physiological processes competing for allocations of that energy^45^,^46^. Lastly, DEB can provide an excellent framework to assess the combined effects from multiple natural and anthropogenic stressors, a topic of greater and greater interest in ecotoxicology and risk assessment.

## Methods

### Model formulation

The basics of the DEBkiss model as well as the underlying assumptions are discussed in detail elsewhere^4^ (https://leanpub.com/debkiss_book). In brief, DEBkiss is a simplified and adapted version of the more complex and parameter heavy standard DEB model, created to more easily implement DEB theory to animals. The major difference with the standard DEB model is the absence of a reserve compartment before separation into the two functional branches (i.e. κ split). The model used here is schematically represented in Fig. 1 and the parameters and their equations are displayed in Table 1. A brief description of the model and the step by step process of mass flow is provided below.

The model here describes the flow of mass in dry weight (though energy can be used) from uptake via feeding and then distributed to fulfil the requirements of physiological processes. Food is processed in the form of assimilates, which are split according to a constant fraction κ which separates the mass flux to somatic maintenance and growth (the soma; κ) and the flux to maturation and reproduction (1 − κ). The scaled functional response *f*, which is the actual feeding rate divided by the maximum feeding rate at that body size (0 = starvation, 1 = *ad libitum* feeding), together with animal surface area and the maximum area-specific assimilation rate (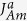) determine the assimilation flux (*J*_*A*_). The fraction of assimilates directed to the soma is prioritized to pay the costs for somatic maintenance (*J*_*M*_) first, and the remainder is used to build structure (e.g. growth; *J*_*V*_). Maintenance here refers to all the costs associated with physiological processes required to maintain body integrity, including protein turnover, muscle activity, maintaining gradients across membranes, etc. The 1 − κ fraction of assimilates is used to fuel maturation, the somewhat abstract term used to describe the costs associated with building the complexity of the animal during development. Maturity maintenance (*J*_*V*_) in this branch is not used to form additional biomass, rather it is consumed to maintain a certain level of complexity and also encompasses the costs associated with immune responses to invading pathogens. Once the animal reaches a maturity threshold (i.e. puberty), maturation ceases and the remainder of the mass flux (after maturity maintenance is paid; *J*_*R.*_) accumulates in a reproduction and supply buffer (RS buffer). The energy in the RS buffer is directly used to fuel reproduction (Table 1). The RS buffer is simply called the ‘reproduction buffer’ in traditional DEB terminology, however we have added ‘supply buffer’ to the term because we view this compartment more generally as an energy storage compartment, comparable to the traditional ‘reserve’. Our interpretation of the RS buffer includes accumulation and storage of surplus energy/mass that can be used for future metabolic needs when the supply of energy is low.

DEBkiss has special rules to dictate the flow of mass when the animal does not assimilate enough energy to cover somatic maintenance costs (i.e. starvation). In this scenario, growth ceases and energy is first diverted from the 1 − κ branch to cover the remaining costs of somatic maintenance; thus under starvation, energy is prioritized for maintenance at the expense of maturation in juveniles or the RS buffer in adults. If energy is still lacking after diverting the 1 − κ branch, maintenance is paid from structure causing decreased body size.

### Model specifications for mink

Body size in our model is defined by structural mass in dry weight (*W*_*V*_), which is directly linked to the volumetric body length (*L*, see Table 1). Volumetric body length is the cubic root of structural volume and is determined using the ratio between structural body mass (*W*_*V*_) and dry weight density (*d*_*V*_) (Table 1).

The simplest DEBkiss model assumes continuous reproduction according to available energy in the RS buffer and does not consider the additional costs of lactation in mammalian reproduction. We have adapted this model to mimic the annual reproductive cycle of the mink. First, we set *t*_p_ as the day when maturation ceases and accumulation of a RS buffer begins; this time threshold was set as a parameter to estimate in the model. Female mink would then produce a litter of kits once a year, the number of which depended on the energy available in the RS buffer. Our simplified reproduction model estimated the reproduction rate *R* as a function of the yield for the conversion of the available RS buffer into embryos as well as the amount of assimilates required to fulfil the costs of embryo formation and cumulative milk production during lactation (see Table 1). Our model assumed pregnancy at Julian day 334, a gestation of 42 days and a 28 day nursing period, such that a complete litter of weaned kits would follow at day 39 of the following year (model day 404) and the process would be repeated every 365 days thereafter. Litter size in captive and wild mink typically range from 3 to 9 kits and milk production varies with litter size and during kit growth^22^,^24^. For simplification, we used the average daily milk production of female mink with litters of six kits; four week cumulative milk production per kit was 597 grams wet weight, equivalent to 131 grams dry weight using a milk dry matter content of 22%^22^. The number of kits produced is therefore determined by the available energy in the RS buffer, in our case modelled to maximize the number of kits rather than a specific litter size. The mass flux used for reproduction is subtracted from the RS buffer, thus resetting this energy reserve for the following year (Table 1).

Female mink increase their food intake during gestation and lactation to cover the energy-intensive costs of reproduction while limiting any fluctuations in their body size^22^,^24^. To address this observed increased feeding during reproduction, we introduced a time dependent adjustment to energy assimilation. During the reproductive cycle (gestation and lactation), we modified the assimilation flux going to reproduction (1 – κ) by replacing the scaled feeding parameter (*f*) with a reproduction specific feeding parameter (*f*_*R*_). The application of *f*_*R*_ only to the 1 – κ branch allowed additional feeding/energy intake to directly fuel reproductive costs while leaving the flux to structure and growth unaltered.

### Modelling toxicant stress

Modelling the effect of toxicant stress in a mechanistic model requires a link between toxicant exposure and primary parameters of the model. This requires a toxicokinetic module to describe toxicant accumulation in the organism. Our model calculates the internal concentration (*C*_*V*_) of toxicants in mink throughout its lifetime based on the level in food while adjusting for growth dilution and maternal offloading to offspring (Table 2). Dietary accumulation was captured using a bioconcentration factor (BCF) defined as the ratio of PCB in adult female mink tissue and their food. The uptake and elimination rate constant *k*_*e*_ is scaled to body size as is the internal concentration in order to account for growth dilution. Reproductive transfer is similarly scaled to body size and is a function of the number of offspring and mass transfer from mother to kit during gestation and lactation.

The toxicodynamics model describes how absorbed toxicants exert their effect on individual model parameters, which in turn alters animal physiology and life-history output over time^4^,^5^. A stress factor or hazard (*S,H*_*C*_) is used to link the internal concentration to some level of effect, which is determined by the level of tolerance (C_*T*_,*C*_*Th*_) above a no-effect concentration (NEC) (Table 2). The effect of toxicants on different DEB parameters can be defined as physiological modes of actions (pMoA) which facilitate our understanding of the ‘target’ site or metabolic process affected by the toxicant. The pMoA and how they are calculated are shown in Table 2. The pMoA are incorporated into the DEBkiss model by replacing a parameter with its associated stressed version. For instance, to simulate the effect of increased somatic maintenance (e.g. repair of lesions caused by a toxicant), 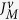 in the calculation of the maintenance flux *J*_*M*_ is replaced with 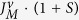. Direct effects on survival were captured for the foetus and newborn kit using the embryo hazard and kit survival pMoA, respectively (Table 2). The kit survival hazard (*H*) is the combination of background mortality and toxicant induced mortality. Kit hazard was converted into a percent survival estimate (%*H*_*s*_), which when multiplied to the reproduction rate provides an estimate of kit survival.

### Experimental data and model calibration

The model was calibrated to body weight measurements of female mink over time^47^. Wet weights were converted to dry weight using a typical dry matter content in vertebrates of 30%^48^. All model calculations were performed with the free modelling software OpenModel 2.4.2 (University of Nottingham, UK). Model parameters were first fit to growth data using the built-in Metropolis-Hastings parameter search in OpenModel, which utilizes a Monte Carlo Markov Chain (MCMC) optimization procedure (see OpenModel User Guide: www.nottingham.ac.uk\environmental-modelling\openmodel.html). Using an average litter size from multiple females of 5.5 kits for the first reproductive event^27^, the parameters were further optimized for the growth and reproductive data together using the same MCMC procedure. These new parameter estimates represented the final values for the baseline (no exposure) mink DEBkiss model (Table 1).

The toxicant stress model was based on mink exposure experiments in which several generations of mink were fed contaminated carp (*Cyprinus carpio*) from Saginaw Bay that contained a mix of organohalogenated contaminants including most notably polychlorinated biphenyls (PCBs)^27^. Mink were fed daily portions of carp formulated to contain either 0 (control), 0.25, 0.5 or 1.0 μg/g ww PCB and body weight and reproductive output was measured in two generations of kits. Here we modelled the growth and survival of the F1 litter (i.e. F2 kits) at the four exposure doses defined above. Litter size and kit survival was measured at birth and again at six weeks of age, and thus these two timepoints were used to model reproductive output in the model. To describe the effect of PCBs, each pMoA was assessed separately by fitting the stress tolerance parameter (*C*_*T*_) and no-effect concentration (*NEC*_*S*_) to the observed data of litter size at birth and growth of kits up to day-42 using the MCMC optimization procedure. The same approach was used for the second reproductive timepoint (litter size at day-42) with additional estimation for the survival hazard tolerance (*C*_*Th*_) and no-effect concentration (*NEC*_*H*_) for kits; here the data was fit to litter size at 6 weeks and therefore combined embryo and kit survival.

All figures were made using the R statistical software^49^ within the ggplot2 graphical package^50^. Reproductive effects as a function of maternal PCB concentrations were described by three parameter log-logistic sigmoid models using the DRC package in R^51^.

## Additional Information

**How to cite this article:** Desforges, J.-P. W. *et al*. Using energy budgets to combine ecology and toxicology in a mammalian sentinel species. *Sci. Rep.* **7**, 46267; doi: 10.1038/srep46267 (2017).

**Publisher's note:** Springer Nature remains neutral with regard to jurisdictional claims in published maps and institutional affiliations.

## Figures and Tables

**Table 1:** DEB parameters and equations used in this paper to model mink growth and reproduction.

Symbol	Description	Equation/value^a^	Unit
Fluxes			
*J*_*A*_	Mass flux for assimilation/feeding	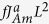	*g/d*
*J*_*M*_	Mass flux for somatic maintenance	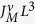	*g/d*
*J*_*V*_	Mass flux for structure	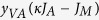	*g/d*
*J*_*J*_	Mass flux for maturity maintenance	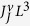	*g/d*
*J*_*R*_	Mass flux to reproduction & supply buffer	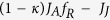	*g/d*
Primary parameters			
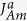	Maximum area-specific assimilation rate	0.24 ± 0.005	g/*cm*^2^*d*
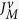	Volume-specific somatic maintenance costs	0.016 ± 0.0001	g/*cm*^3^*d*
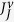	Volume-specific maturity maintenance costs	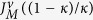	g/*cm*^3^*d*
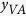	Yield of structure on assimilates	0.80 ± 0.000	*g/g*
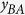	Yield of reproduction & supply buffer on assimilates	0.95^b^	*g/g*
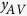	Yield of maintenance on assimilates	0.80 ± 0.000	*g/g*
*κ*	Fraction of assimilation flux to soma	0.80^b^	—
*W*_*B*_	Mass of assimilates in a newborn kit	2.55^c^	*g*
*W*_*M*_	Mass of assimilates produced during lactation per kit	131^d^	*g*
Other parameters			
*f*	Scaled functional response	1.00	—
*f*_*R*_	Scaled functional response during reproduction	2.52 ± 0.092	—
*L*	Volumetric body length	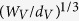	*cm*
*L*_*m*_	Maximum volumetric body length	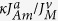	*cm*
*d*_*V*_	Dry weight density	0.30^b^	g/cm^3^
*R*	Reproduction rate	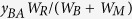	#/*d*
State variables			
*W*_*V*_	Mass of structural body 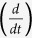	*J*_*V*_	*g*
*W*_*R*_	Mass of assimilates in reproduction & supply buffer 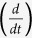	*J*_*R*_	*g*
*R*_*C*_	Cumulative reproduction rate 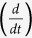	*R*	#

^a^Fitted parameters are presented by their determined values ± standard deviations.

^b^Default values in DEB theory^48^.

^c^Calculated from weight wet of kits at birth^27^.

^d^Calculated from four week wet weight cumulative milk production^22^.

**Table 2:** Stress functions and DEB parameters affected by toxicants through different physiological modes of actions.

Symbol	Description	Equation	Units	Affected parameter
*S*	Stress factor	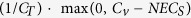	/*d*	
*H*	Survival hazard	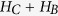	/*d*	
*H*_*C*_	Survival hazard from toxicant	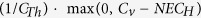	/*d*	
*H*_*B*_	Background survival hazard at t_42_	0.25^a^	/*d*	
%*H*_*S*_	Percent survival at t_42_	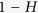	*%*	
*NEC*_*S*_	No effect concentration for the stress factor	0.017 – 0.65^b^	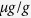	
*NEC*_*H*_	No effect concentration for the survival hazard	9.12 × 10^−6^ – 1.08 × 10^−5b^	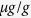	
*C*_*V*_	Internal toxicant concentration^a^	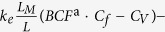	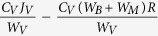	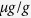
*k*_*g*_	Elimination rate constant	0.0029 ± 0.001	/*d*	
*C*_*T*_	Stress tolerance	0.69 − 2.25^b^	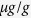	
*C*_*Th*_	Survival hazard tolerance	2.94 − 3.71^b^	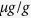	
Physiological modes of action				
**Feeding/assimilation**	Decreased assimilation	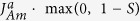	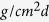	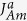
**Somatic maintenance**	Increased somatic maintenance	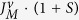	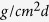	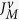
**Growth cost**	Increased overhead cost of growth	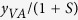	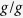	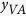
**Reproduction cost**	Increased cost per embryo	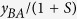	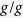	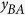
**Embryo hazard**	Decreased embryo survival	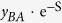	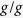	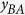
**Kit survival**	Decreased kit survival at t_42_	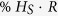	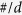	*R*

^a^Values taken from empirical observations^27^. BCF is the bioconcentration factor from the diet (fish) to mink liver. Initial value of 1.8 was taken from empirical observations^27^ and final value was estimated using MCMC fitting to the data.

^b^Range of values determined from fitting to data for each individual pMoA being tested.
